# High-fat diet decreases the expression of Kiss1 mRNA and kisspeptin in the ovary, and increases ovulatory dysfunction in postpubertal female rats

**DOI:** 10.1186/1477-7827-12-127

**Published:** 2014-12-26

**Authors:** Qiangyong Zhou, Haiyan Chen, Simeng Yang, Yuehua Li, Binqiao Wang, Yuanyuan Chen, Xueqing Wu

**Affiliations:** Department of Obstetrics and Gynecology, The First Affiliated Hospital of Wenzhou Medical University, Wenzhou, Zhejiang China

**Keywords:** High-fat diet, Kiss1, Kisspeptin, Oestrous cycle, Ovulation

## Abstract

**Background:**

Female reproductive health is noticeably compromised by obesity. The underlying mechanisms remain to be elucidated. Accumulating evidence indicates that the expression level of ovarian *Kiss1* peaks in the afternoon during prooestrus, suggesting local regulatory roles for *Kiss1* in the ovulatory process. We used a diet-induced model of obesity to evaluate whether the ovarian *Kiss1* system is affected by obesity, and, to investigate the association of the *Kiss1* system with ovulatory disorders in female rats.

**Methods:**

Post-weaning, female, Sprague–Dawley rats were randomly fed either a high-fat diet (HFD) or a normal chow diet (NCD) until they reached postnatal day 30 (PND 30), PND 42, or PND 70. The timing of vaginal opening was recorded, and oestrous cyclicity was monitored for 2 consecutive weeks immediately post puberty and again at 8–9 weeks of age. Tissues from the left ovary were collected for determination of the levels of *Kiss1* and *G protein-coupled receptor 54* (*GPR54*) mRNA, and tissues from the right ovary were collected for assessment of the immunoreactivity (IR) of the corresponding protein products, kisspeptin and GPR54.

**Results:**

The high-fat diet resulted in a significantly higher body weight and an earlier puberty onset. Oestrous cyclicity was disrupted by the HFD with significant reductions in the expression of ovulation-related genes. A marked suppression of ovarian *Kiss1* mRNA levels was observed during prooestrus and oestrus at PND 42, and, during prooestrus, oestrus, and metoestrus at PND 70 in the HFD rats compared with the NCD controls. In the HFD group, the immunoreactivity of kisspeptin was significantly lower in theca cells from antral follicles during prooestrus and oestrus at PND 42, and, during prooestrus, oestrus at PND 70. At the prooestrus stage, in the HFD group the immunoreactivity of kisspeptin was also lower in the theca cells of preovulatory follicles at both PND 42 and PND 70.

**Conclusions:**

Exposure of female rats to an post-weaning, high-fat diet has long-term deleterious effects on ovulation, that may involve down-regulation of ovarian *Kiss1* mRNA and kisspeptin.

## Background

The prevalence of obesity continues to rise, presenting a significant public health concern [1, 2]. Epidemiological studies show that obesity increases the risk of cardiovascular disease, type-II diabetes, hypertension, cancer and metabolic disorders [2], as well as reproductive dysfunction [3–6]. There is a clear association between energy balance and reproduction, and, excess energy intake frequently results in fertility impaired in both men and women [3–6]. In women, obesity has detrimental effects on conception and implantation [4], as well as foetal development [5], as well as inducing anovulation and menstrual abnormality [6]. Additionally, obesity is associated with premature puberty in girls [6], and this effect has also been described in animal models of obesity [7, 8]. However, the mechanisms underlying the adverse reproductive consequences of obesity remain to be elucidated.

Kisspeptins, a family of structurally related peptides that are encoded by the *Kiss1* gene and bind to the G protein-coupled receptor GPR54, have emerged as a critical upstream regulator of the hypothalamic-pituitary-gonadal (HPG) axis [9]. The hypothalamic *Kiss1* system, consisting of two neuron populations located in the anteroventral periventricular nucleus (AVPV) and the arcuate nucleus (ARC) in rodents, is fundamental to fertility through its regulation of the secretion of gonadotropin-releasing hormone, and it plays an important role in pubertal maturation and the attainment of reproductive function [9, 10]. In AVPV, the level of *Kiss1* mRNA has been shown to be highest during proestrous and lowest during metoestrus. Besides, the level of *Kiss1* mRNA in ARC was highest during dioestrus and lowest during proestrous [10]. Furthermore, regional- and cycle-specific expression of *Kiss1*/*GPR54* has been documented in the ovaries in various species. Ovarian *Kiss1* expression peaks in the afternoon during prooestrus, suggesting local regulatory roles for kisspeptin in the ovulatory process [11, 12]. This hypothesis is also supported by the demonstration of marked suppression of ovarian *Kiss1* mRNA levels during the ovulatory period in a model of ovulatory dysfunction induced by administration of indomethacin [13].

In recent years, mounting evidence has suggested that kisspeptins, at least in part, represent a link between energy status and reproduction. Analyses in both pubertal and adult female rats, subjected to energy insufficiency, demonstrated a significant decrease in *Kiss1* expression in the hypothalamus, with a detectable reduction in LH levels [14, 15]. Exogenous administration of kisspeptins can rescue the gonadotropic dysfunction associated with the above-mentioned conditions [14]. Likewise, female DBA/2 J mice that were maintained on a high-fat diet, presented a marked decrease in *Kiss1* mRNA levels in both the ARC and the AVPV, as well as a decrease in the number of kisspeptin-expressing neurons in the AVPV compared with chow-fed controls [16]. However, considering the potential direct effects of the *Kiss1* system on the ovaries, it remains to be determined whether ovarian *Kiss1* is involved in the impaired reproductive function in obese individuals, especially during ovulation. Using a diet-induced model of obesity, the aim of this study was to evaluate the influence of obesity induced by a high-fat diet on the ovarian *Kiss1* system and the ovulatory capacity in postpubertal rats.

## Methods

This study was approved by the Laboratory Animal Ethics Committee of Wenzhou Medical University in April 2011 (NO. wydw2011-001).

### Animals and diets

Pregnant, female, Sprague–Dawley rats (Slac) were housed individually under constant conditions of light (12-hour light–dark cycle, with light on at 07:00) and temperature (222°C), with food and water available *ad libitum*. On the day of birth (PND 0), the pups were weighed and sexed, and the litter size was adjusted to twelve pups per litter on PND 1 to ensure equal nutrition and care until weaning (PND 21). On PND 21, the female pups were randomly divided into the HFD and NCD groups, which were balanced for the body weights of the pups. In the prepubertal groups, the HFD rats were fed a high-fat diet (40% kcal as fat; Slac) until PND 30, while the NCD rats were fed a normal chow diet (5% kcal as fat; Slac). In the postpubertal groups, the HFD and NCD rats were exposed to a high-fat diet or a normal chow diet, respectively, until PND 42 and PND 70. The animals were housed four to six per cage and weighed weekly until the end of the experiment. All experiments were approved by the Animal Care and Use Committee of Wenzhou Medical University and were in accordance with the National Research Council's Guide for the Care and Use of Laboratory Animals.

### Assessment of puberty onset and oestrous cyclicity

Beginning on PND 28, the animals were monitored daily for vaginal opening, which is a physical marker of the onset of puberty. Once vaginal opening occurred, the body weight was recorded and vaginal smears were performed daily at 08:00 h to determine the oestrous cyclicity for 14 consecutive days, and again from PND 56 to PND 70. The oestrous stage (prooestrus, oestrus, metoestrus, or dioestrus) was determined according to the relative abundance of nucleated vaginal epithelial cells, cornified epithelial cells and leukocytes in the vaginal smears under a light microscope. A normal oestrous cycle was defined as including at least two, consecutive, regular, oestrous cycles, lasting for 4–5 days with 1–2 days of oestrus [17].

### Tissue processing

At the end of each study, the animals were deeply anaesthetised with 10% chloral hydrate (3 mg/100 g, i.p.; Sigma-Aldrich) in the afternoon, and they were decapitated immediately after the vaginal smears were performed. Two ovaries were rapidly removed from each rat and dissected out of the surrounding fat pad. One ovary was fixed in 4% paraformaldehyde for later immunohistochemical staining, while the other was snap frozen on dry ice and stored at −80°C for RNA analysis.

### Real-time RT-PCR

Total RNA was isolated from the ovarian samples using TRIzol reagent (Invitrogen) according to the manufacturer’s instructions. Reverse transcription was carried out using a First Strand cDNA Synthesis Kit (Thermo) in a total volume of 20 μl according to the manufacturer's protocols. The resultant cDNA was amplified using a SYBR Green qPCR kit (Toyobo), and it was quantified using an ABI PRISM 7500 sequence detection system (Applied Biosystems). The specific primer details are shown in Table 1. The PCR reaction mixture consisted of 2 μl of cDNA, 0.4 μl each of specific primers (10 μM) and 6 μl SYBR Green Mix in a total volume of 10 μl. The PCR cycling conditions for *Kiss1*, *GPR54,* four genes related to follicular growth and ovulation (*growth differentiation factor 9*, *GDF9*; *bone morphogenetic protein 15*, *BMP15*; *follicle stimulating hormone receptor*, *FSHR*; *prostaglandin-endoperoxide synthase 2*, *PTGS2* [also known as *COX2*]), and *Hypoxanthine phosphoribosyltransferase 1* (*HPRT1*) were as follows: an initial denaturation and activation at 95°C for 5 min, followed by 40 amplification cycles of 95°C for 20 s and 60°C for 60 s. The mRNA levels of the target genes were normalised relative to the reference gene *HPRT1* as previously described [7]. A melting-curve analysis for each PCR product showed that single products were amplified, and this was confirmed by gel electrophoresis. The relative expression levels of each gene were obtained using the comparative Ct method as described previously [18] and are depicted as the fold change in relation to the control group.

**Table 1:** **Primer sequences used in the qPCR experiments**

Gene name	Primer sequences (5’–3’)	Expected size(bp)	Accession Number (GenBank)
*Kiss1*	Forward TGCTGCTTCTCCTCTGTGTGG	110	NM_181692.1
	Reverse ATTAACGAGTTCCTGGGGTCC		
*Kiss1r*	Forward CTTTCCTTCTGTGCTGCGTA	102	NM_023992.1
	Reverse CCTGCTGGATGTAGTTGACG		
*GDF9*	Forward GAGGAAACACAGGTGGATTGAGATG	174	NM_021672.1
	Reverse AGATACAAGATGAGCGAGGGCG		
*BMP15*	Forward GCTAAAATGGTGAGGCTGATAAA	180	NM_021670.1
	Reverse ATGGCAGGAGAGATGGTAATG		
*FSHR*	Forward CTACACATTGACAGCCATCACCCTA	121	NM_199237.1
	Reverse GGCAAAAGTCCAGCCCAATACC		
*PTGS2*	Forward TCTCCAACCTCTCCTACTACACCA	139	NM_017232.3
	Reverse ATGAACTCTCTCCTCAGAAGAACCT		
*HPRT1*	Forward CCCCAAAATGGTTAAGGTTGC	175	NM_012583.2
	Reverse TCCACTTTCGCTGATGACACAA		

### Immunohistochemistry analysis

Ovarian tissues from postpubertal female rats at different stages of the oestrous cycle were fixed overnight in 4% paraformaldehyde at 4°C and routinely processed for paraffin embedding. Histological sections (5 μm) were then prepared for further analysis. For kisspeptin/GPR54 immunohistochemistry, the sections were mounted on poly-L-lysine-coated slides. After deparaffinisation in xylene and rehydration through decreasing concentrations of ethanol, the slides were transferred to sodium citrate buffer for antigen retrieval in a microwave oven (2*10 min, 20 power). The slides were rinsed in PBS, followed by incubation in 1% hydrogen peroxide for 30 min to quench the endogenous peroxidase activity, and blocked with 10% non-immune goat serum for 15 min at room temperature. Thereafter, the sections were incubated overnight with a primary rabbit anti-kisspeptin antibody (1:300 dilution; product no. 251265; Abbiotec) or a rabbit anti-GPR54 antibody (1:100 dilution; product no. 254512; Abbiotec) at 4°C in a moist chamber. The sections were then washed in PBS and incubated with biotinylated goat anti-rabbit secondary antibodies (Zhongshan Goldenbridge) for 1 h at room temperature, followed by incubation with a conjugated avidin–biotin complex (Zhongshan Goldenbridge) for an additional 30 min at room temperature according to the manufacturer's instructions. The kisspeptin/GPR54 immunoreactivity was visualised with 3,3′-diaminobenzidine tetrahydrochloride (DAB) solution (Sigma-Aldrich). Finally, the slides were counterstained with haematoxylin, dehydrated, and mounted. For negative controls, adjacent sections were processed without a primary antibody. Immunohistochemical detection of the rat oviduct was included as a positive control [19]. The sections were analysed on a light microscope (Olympus). To study the relationship between the *Kiss1* system and ovulation, antral follicles at each oestrous stage and preovulatory follicles at prooestrus were included in the immunohistochemistry analysis. The follicles were classified as previously reported [17]. For each antibody, two follicles per section and three sections per ovary were included in IHC analysis. Semi-quantitative evaluation of the immunostaining intensity was performed using Image-Pro Plus 6.0 system as described previously [20], and mean optical density (MOD) was used to indicate the level of kisspeptin/GPR54 expression.

### Statistics

The results were expressed as the means ± SEMs, and the analyses were performed using the Statistical Package for the Social Science (SPSS) 17.0 software. The comparisons between the HFD and NCD groups regarding body weight, puberty onset, the level of *Kiss1, GPR54, GDF9, BMP15, FSHR, PTGS2* mRNA, and the immunostaining intensity of kisspeptin/GPR54 were statistically analysed using a two-sample t-test. The relative abundance between different oestrous stages within an experimental group was compared by a one-way analysis of variance (ANOVA). The effect of HFD on the regularity of the oestrous cycle was tested using the Chi-squared test. P values <0.05 were considered statistically significant.

## Results

### Effect of HFD exposure on body weight and puberty onset

**Figure 1:**
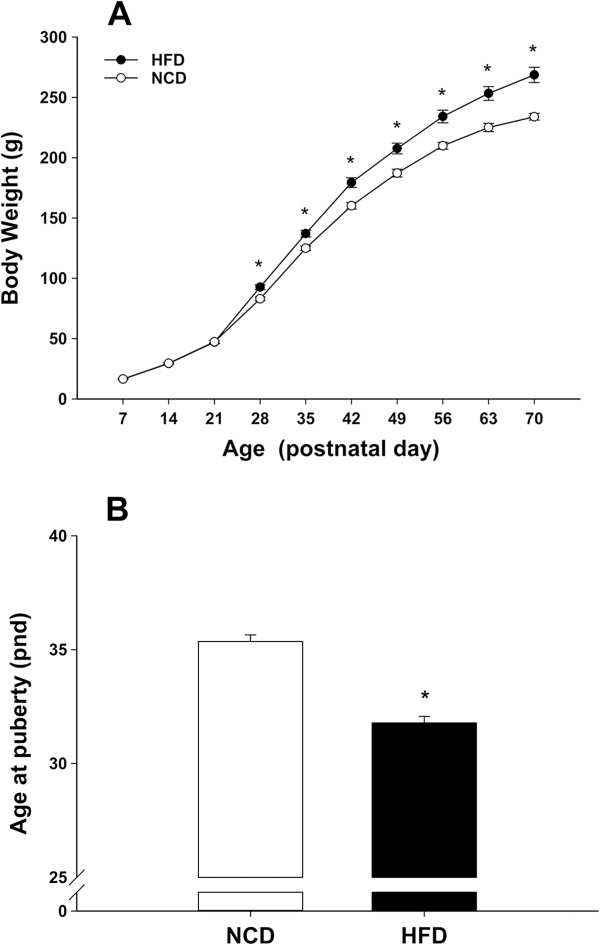
**Effects of postweaning exposure to a HFD on body weight and the onset of puberty. A**, HFD exposure significantly increased body weight from PND 21 onward compared with exposure to the normal control diet; **B**, The age at puberty onset was significantly advanced in rats fed with the HFD. *P < 0.05, versus NCD controls (n = 18–38 for each group).

Before the dietary treatment, the mean body weights ± SEMs for the HFD and NCD groups were not significantly different (HFD: 48.3 ± 0.8 vs NCD: 47.8 ± 0.9; Figure 1A; P > 0.05). At 6 weeks of age (PND 42), the HFD group had a significantly higher body weight compared with the controls (HFD: 179.4 ± 4.0 vs NCD: 160.2 ± 2.7; Figure 1A; P < 0.05). Similarly, feeding an HFD further increased the body weight at 10 weeks of age (PND 70) compared with the controls (HFD: 268.7 ± 6.4 vs NCD: 234.0 ± 2.8; Figure 1A; P < 0.05). Exposure of the animals to the HFD resulted in a significant advance in the onset of puberty (HFD: 31.8 ± 0.3 vs NCD: 35.4 ± 0.3; Figure 1B; P < 0.05).

### Effects of HFD exposure on oestrous cyclicity and expression of ovulation-related genes

**Figure 2:**
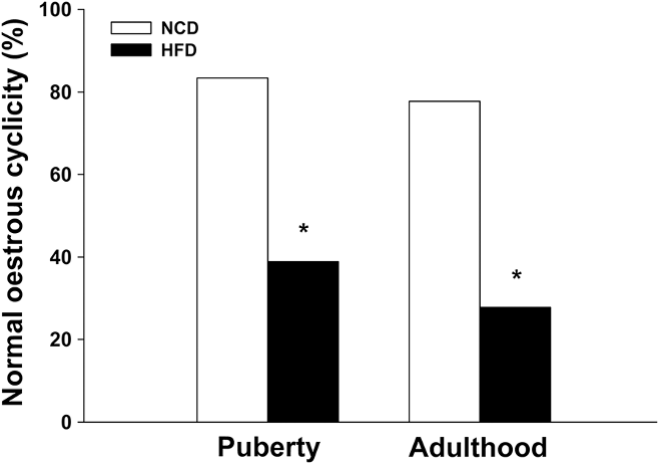
**Effects of postweaning HFD exposure on oestrous cyclicity in pubertal and adult rats.** Female rats fed with a HFD postweaning are more likely to have an irregular oestrous cyclicity. *P < 0.05, versus NCD controls (n = 18 for each group).

**Figure 3:**
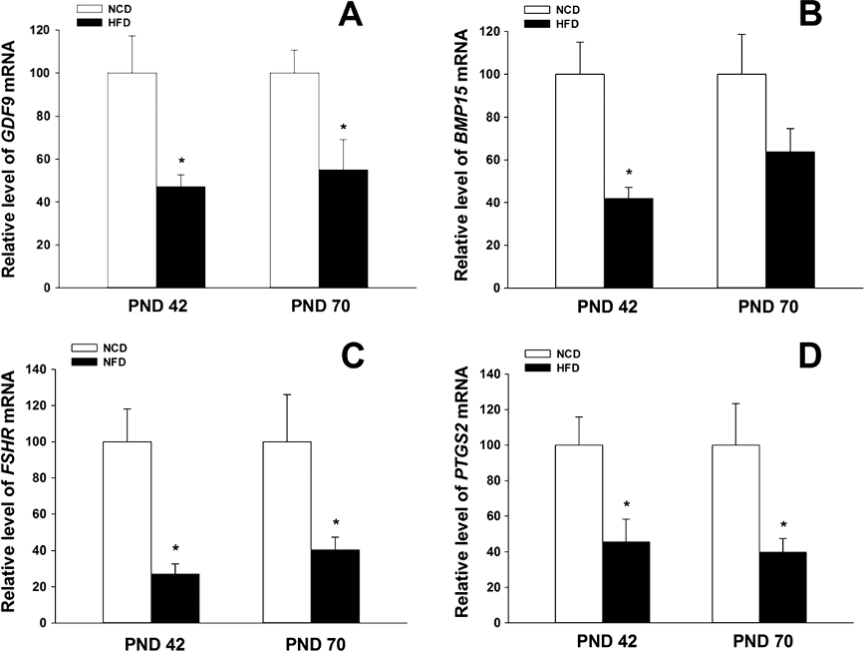
**Effects of HFD exposure on the expression of genes related to follicular growth and ovulation.** There were significantly lower levels of *GDF9*, *FSHR*, and *PTGS2* mRNA during prooestrus at both PND 42 and PND 70, in the HFD groups compared with the NCD groups **(A**, **C** and **D)**, whereas the expression of BMP15 only showed significant difference at PND 42 **(B)** . *P < 0.05, versus NCD controls (n = 6 for each group)

Post-weaning HFD exposure had a negative effect on oestrous cyclicity (Figure 2). In puberty, the majority (83.3%) of the NCD controls had normal 4–5-day oestrous cycles, whereas only 38.9% of HFD rats showed regular cyclicity. This disruption was also demonstrated in adulthood, with 77.8% of the NCD rats showing normal cyclicity compared to only 27.8% in the HFD group. The HFD rats had a significantly lower percentage of normal cyclicity during both puberty and adulthood compared with the NCD controls. At late prooestrus, expression levels of *GDF9*, *FSHR* and *PTGS2* in the HFD rats were lower at both PND 42 and PND 70 (Figure 3A, C and D), whereas the expression of *BMP15* only showed significant difference between the two groups at PND 42 (Figure 3B).

### Effects of HFD exposure on expression of *Kiss1*/*GPR54*

**Figure 4:**
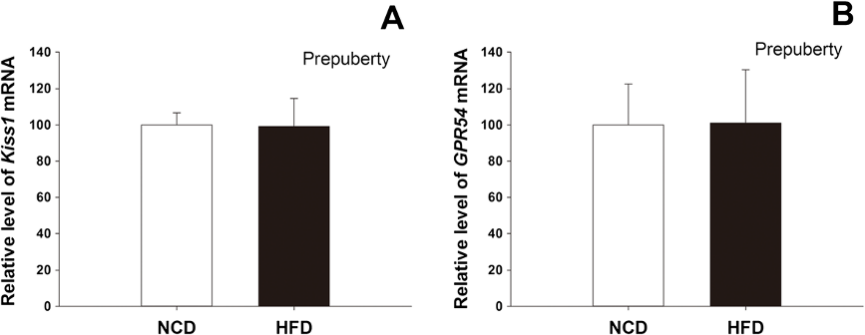
**Effects of postweaning HFD exposure on the expression of** ***Kiss1*** **and** ***GPR54*** **in prepubertal rats.** At PND 30, there was no significant difference in the expression of ovarian *Kiss1* **(A)** and *GPR54* **(B)** mRNA between the HFD and NCD rats (n = 6 for each group).

**Figure 5:**
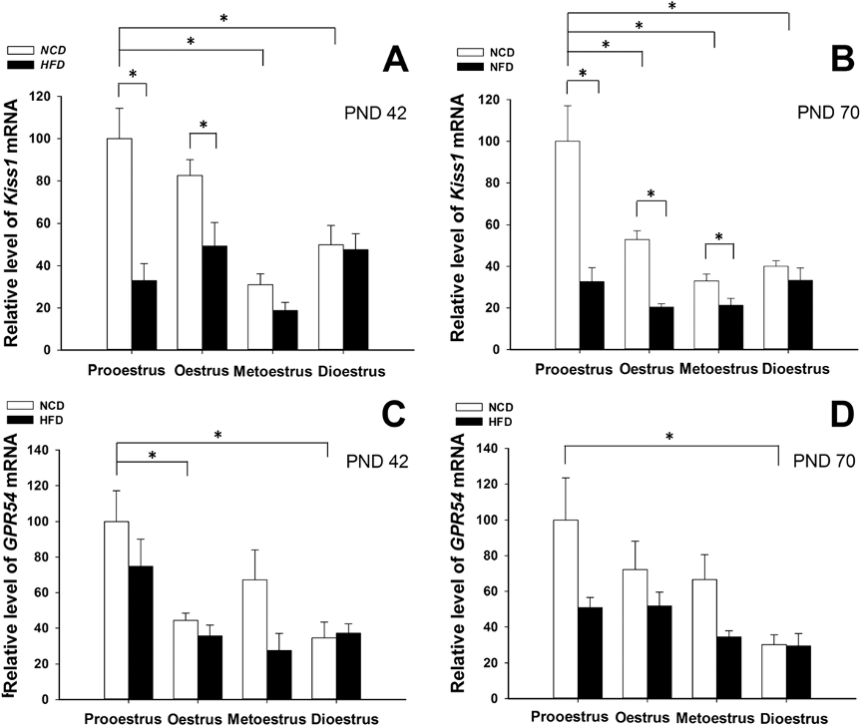
**Effects of postweaning HFD exposure on the expression of** ***Kiss1*** **and** ***GPR54*** **in postpubertal rats.** There were significantly lower levels of *Kiss1* mRNA during prooestrus and oestrus at PND 42 **(A)** and during prooestrus, oestrus and metoestrus at PND 70 **(B)** in the HFD group compared with the NCD group, although the *GPR54* mRNA levels were not different **(C** and **D)**. Within the NCD groups at each oestrous stage, the expression levels of *Kiss1* and *GPR54* mRNA were relative high during prooestrus at both PND 42 and PND 70. *P < 0.05. n = 4–6 for each group.

The expression of *Kiss1* and *GPR54* in the ovary was analysed in rats fed the HFD or the NCD. The results are shown in Figures 4 and 5. At PND 42, significant reductions in *Kiss1* expression were detected at prooestrus and oestrus in the ovaries of the HFD rats compared with the NCD rats (Figure 5A). Similar suppression of *Kiss1* mRNA was also observed in the HFD rats at PND 70, with a significant reduction at prooestrus, oestrus and metoestrus (Figure 5B). These data demonstrate the down-regulation of the *Kiss1* gene in the HFD rats during follicle maturation and ovulation. In contrast, administration of the HFD did not significantly alter the *GPR54* mRNA expression compared to the controls at any stage of the oestrous cycle at either PND 42 or PND 70 (Figure 5C and D). At both PND 42 and PND 70, the expression levels of *Kiss1* and *GPR54* mRNA were relative high during prooestrus within the NCD groups at different oestrous stage. In prepubertal groups, there was no significant difference of ovarian *Kiss1* and *GPR54* expression between the HFD and NCD rats (Figure 4).

### Effects of post-weaning HFD exposure on the immunoreactivity of kisspeptin/GPR54

**Figure 6:**
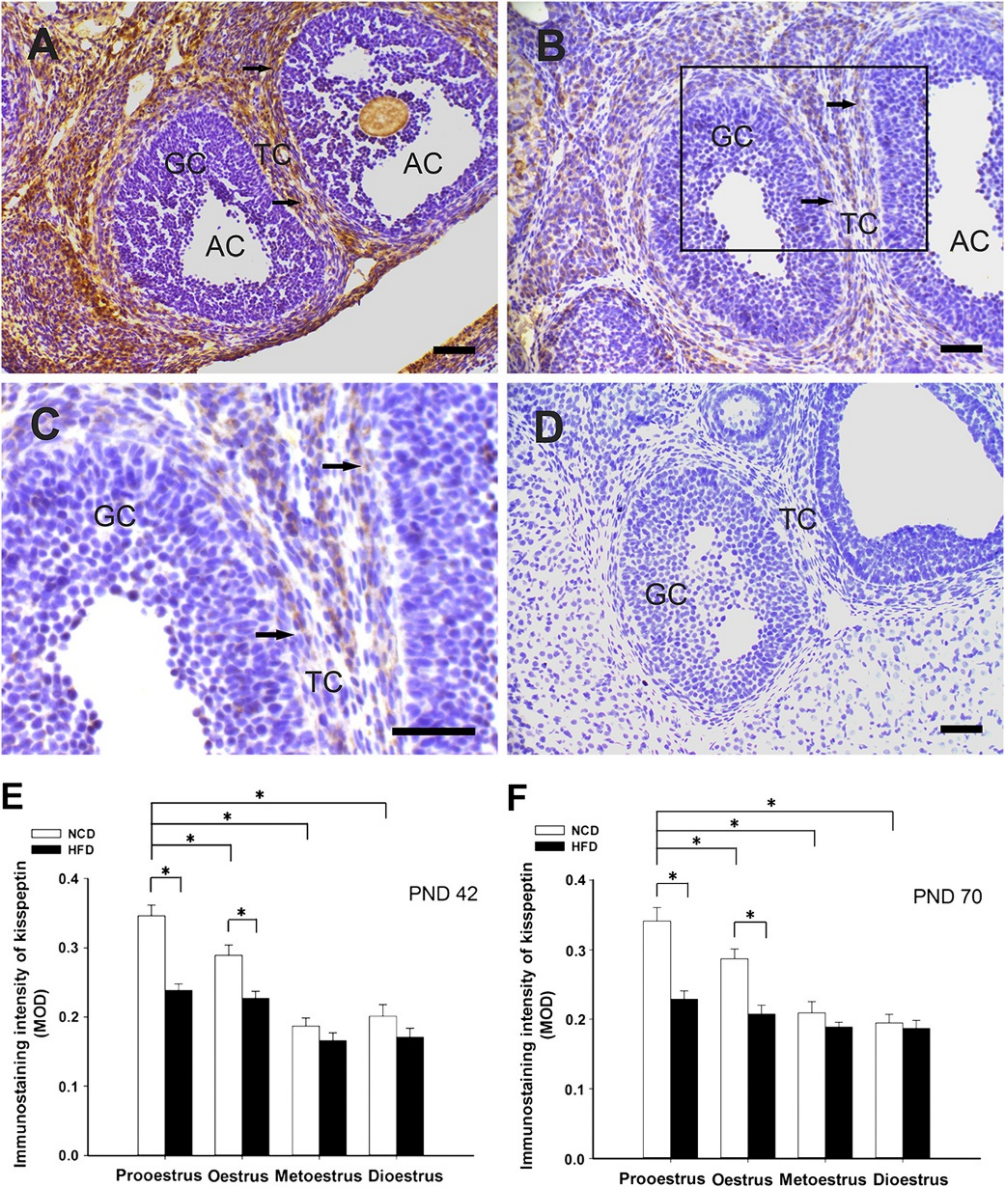
**Effects of exposure to a HFD on kisspeptin-IR in the theca cells of antral follicles.** Kisspeptin-IR was detected in the theca cells (arrows) of antral follicles from the NCD controls **(A).** A representative section shows that the level of kisspeptin-IR in the theca cells of the antral follicles was significantly lower in the HFD-fed rats **(B)**. **(C)** A higher magnification of the boxed area in **(B)**. Immunostaining was absent in sections from the negative controls **(D)**. The MOD for kisspeptin in the theca cells are summarised in **(E)** and **(F)**. AC, antral follicle; GC, granulosa cell; TC, theca cell. Photomicrographs illustrate the prooestrus stage of oestrous cycle at PND 42. Scale bar = 50 μm. *P < 0.05. n = 4–6 for each group.

**Figure 7:**
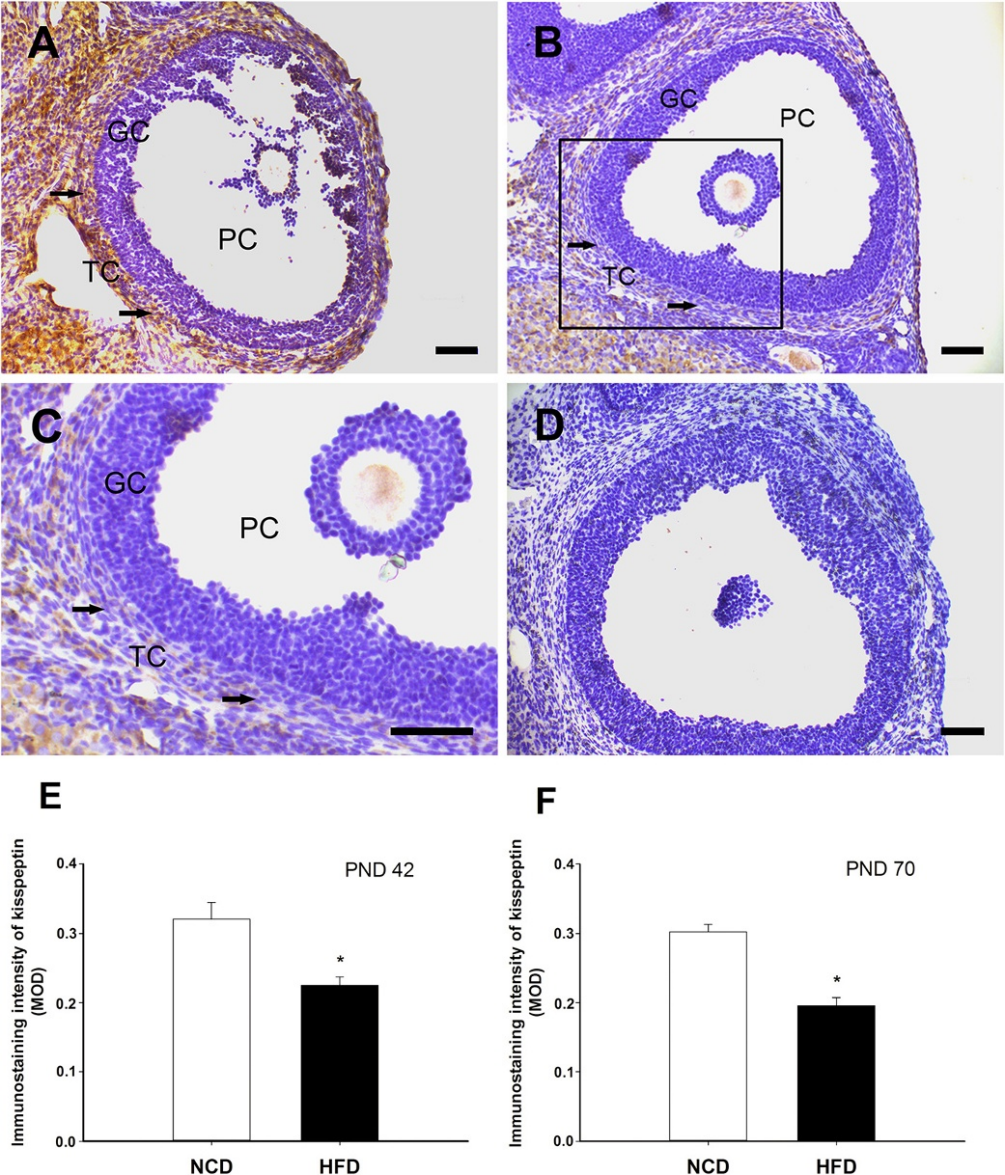
**Effects of exposure to an HFD on kisspeptin-IR in the theca cells of preovulatory follicles.** At the prooestrus stage, kisspeptin-IR was detected in the theca cells (arrows) of preovulatory follicles from NCD controls **(A)**. The level of kisspeptin-IR in the theca cells of preovulatory follicles was significantly lower in the HFD-fed rats **(B). (C)** A higher magnification of the boxed area in **(B)**. Immunostaining was absent in sections from negative control rats **(D)**. The MOD for kisspeptin in the theca cells are summarised in **(E)** and **(F)**. GC, granulosa cell; PC, preovulatory follicle; TC, theca cell. Photomicrographs illustrate the prooestrus stage of the oestrous cycle at PND 42. Scale bar = 50 μm. *P < 0.05, versus NCD controls (n = 4–6 for each group).

**Figure 8:**
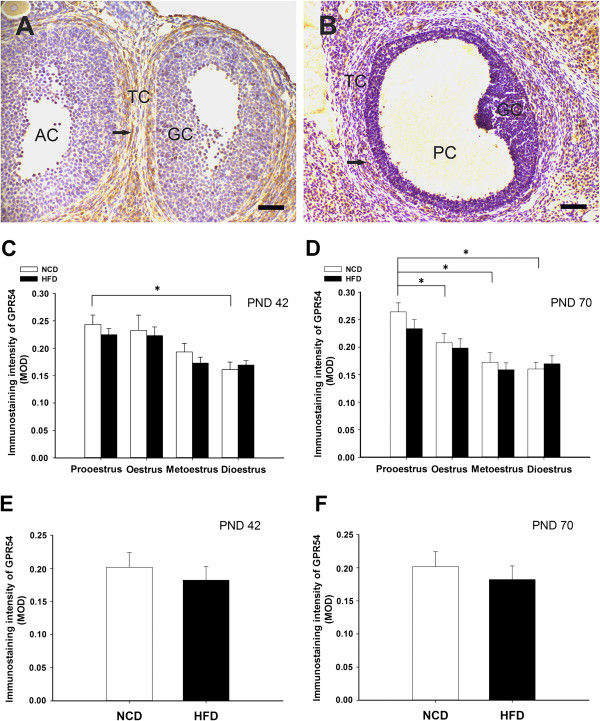
**Effects of postweaning HFD exposure on GPR54-IR in theca cells of antral and preovulatory follicles.** GPR54-IR was detected in the theca cells (arrows) of antral **(A)** and preovulatory follicles **(B)** in NCD controls. The MOD for GPR54 in the theca cells are summarised in **(C)**, **(D)**, **(E)** and **(F)**. At both PND 42 and PND 70, there was no significant difference in the immunostaining intensity in the antral **(C, D)** and preovulatory **(E, F)** follicles between the HFD and NCD groups. AC, antral follicle; GC, granulosa cell; PC, preovulatory follicle; TC, theca cell. The photomicrographs illustrate the prooestrus stage of the oestrous cycle at PND 42. Scale bar = 50 μm. *P < 0.05. n = 4–6 for each group.

In the ovaries of the NCD rats, kisspeptin and GPR54-IR were detected in both the antral and preovulatory follicles. Although there was some staining for kisspeptin/GPR54 in the granulosa cells, the staining was mainly detected in the theca cells (kisspeptin: Figure 6A, Figure 7A; GPR54: Figure 8A and B). The mean optical density in the HFD groups indicated that the levels of kisspeptin expression in the theca cells of the antral follicles were significantly lower during prooestrus and oestrus at PND 42, and during prooestrus, oestrus at PND 70 (Figure 6E and F). In addition, kisspeptin expression was also suppressed by the HFD in the theca cells of preovulatory follicles from prooestrus ovaries compared to controls (Figure 7E and F). However, there was no significant difference in the staining intensity of GPR54 between the HFD and NCD groups in the antral and preovulatory follicles (Figure 8C-F). At both PND 42 and PND 70, the mean optical density for kisspeptin and GPR54 in theca cells of antral follicles, were relative high during prooestrus within the NCD groups at different oestrous stage (Figure 6E and F; Figure 8C and D).

## Discussion

In the present study, we investigated the influence of obesity at two time points following HFD exposure: at PND 42 and at PND 70 in female rats. Post-weaning, HFD exposure was effective in promoting obesity in female rats, as demonstrated by the higher body weight at the end of treatment. In addition, puberty was advanced and oestrous cyclicity was disrupted with significant reductions in the expression of ovulation-related genes. There was a marked suppression of ovarian *Kiss1* mRNA expression and kisspeptin immunoreactivity in the theca cells in antral and preovulatory follicles at the ovulatory transition stage of the oestrous cycle (prooestrus and oestrus) in the rats exposed to a HFD at both PND 42 and PND 70. This result supports the hypothesis that, in addition to its role in the central nervous system, the *Kiss1* system may affect ovulation by acting through peripheral pathways in obese individuals. The present study demonstrates differential gene and protein expression of ovarian *Kiss1* between obese and normal rats in the postpubertal phase. Compared to other ovarian compartments, kisspeptin in granulosa cells is directly involved in the process of ovulation.

Postnatal undernutrition delays the onset of puberty in female rats [21]. In the current study, postweaning HFD exposure resulted in a higher, body weight gain and a significant advancement in the onset of puberty. Early onset of puberty has also been described in obese rabbits [22]. Moreover, in rhesus monkeys, administration of a high-calorie diet resulted in an elevated body mass index and precocious menarche [8]. In humans, menarche occurred significantly earlier in overweight/obese girls (12.5 years) compared with normal weight (12.9 years) and underweight girls (13.7 years) [23]. The issue of an early age at menarche has recently received attention because of the associated increased risk of clinical complications later in life, including asthma, cardiovascular disease, diabetes, and breast cancer [24]. In addition, an earlier puberty may have an impact on reproductive capacity by reducing the functional ovarian reserve [25], and it may be associated with an earlier age at menopause [26].

Although the mechanisms through which obesity affects the timing of puberty remain unclear, central kisspeptin may be an essential gatekeeper of the onset of puberty because genetic inactivation of *Kiss1* or its receptor results in a congenital gonadotropin deficiency and impaired puberty progression [9]. Comparative analyses of physiological changes in response to HFD in female rats demonstrated that advanced puberty induced by a HFD is associated with an accelerated LH pulse frequency and up-regulation of central kisspeptin expression [7]. Similarly, exposing female rabbits to a high-fat, high-cholesterol diet also resulted in a significantly higher LH response and an early onset of puberty [22]. The upstream excitatory effect of kisspeptin on gonadotropin secretion may explain these observations [9]. In contrast, another study reported that postnatal HFD exposure had no effect on puberty onset or central kisspeptin expression [27]. Furthermore, overfeeding in female rats, a model of obesity induced by small litter size, significantly affects puberty onset without having a detectable effect on hypothalamic kisspeptin [28]. The differences in these studies may be the result of the fat content of the food and the timing of the intervention. Rats are able to reduce their food intake when the fat content is too high [29]. In addition, preweaning exposure to overnutrition may induce postweaning catch-up growth [28]. Direct ovarian administration of a kisspeptin antagonist in prepubertal rats resulted in delayed vaginal opening, thus indicating that ovarian kisspeptin participates in the initiation of female puberty [30]. However, in our model of early puberty, we demonstrated that there is no significant difference in the expression level of ovarian *Kiss1* and *GPR54* mRNA between HFD and NCD rats in the prepubertal period. Considering the dominant role of central kisspeptin in puberty onset that has been widely recognized, we believe that the action of ovarian kisspeptin on puberty onset might be modulatory. The potential mechanism needs to be further elucidated.

Our present results demonstrate that female rats exposed to a HFD had significantly irregular oestrous cyclicity during both puberty and adulthood, a phenomenon that represents ovulatory dysfunction. Moreover, the analysis of the genes related to follicular growth and ovulation show that these genes were down-regulated at late prooestrus, a phase during which follicles grow and mature for ovulation. Although notable differences exist in the process of ovulation, a key event preceding ovulation is a surge in the LH levels resulting from positive feedback from oestradiol [31]. Related endocrine changes have been observed in obesity-induced ovulatory dysfunction in many species. Female rats fed on a high-fat diet (45% calories from fat) lacked the expected LH surge and exhibited a reduction in the plasma oestradiol levels on the day of prooestrus [32]. In rabbits, HFD exposure negatively affects ovulation by reducing the peak LH concentrations in response to GnRH stimulation at 22 weeks of age [22]. In humans, female obesity was linked to a dramatic reduction in both the maximal LH level and the mean LH level compared to controls [33]. These data are suggestive of down-regulation of hypothalamic *Kiss1* signalling. Analyses in obese DBA/2 J mice, which are prone to HFD-induced fertility problems, show a marked decrease in *Kiss1* expression in both the AVPV and ARC, as well as in the number of kisspeptin-IR cells in the AVPV [16]. Altogether, these observations support the hypothesis that energy stress could suppress the LH surge, inhibit normal ovulation, and disrupt oestrous cyclicity through functional impairment of the central *Kiss1* system.

In parallel to the analyses of the central effects of the *Kiss1* system on reproductive maturation and function, some data suggest additional functions of the *Kiss1* system in peripheral organs [11, 12]. To test this possibility, we measured the immunoreactivity of kisspeptin/GPR54 in rat ovaries. In accordance with a previous study [11], our data show that kisspeptin/GPR54 immunoreactivity is detected in different ovarian compartments, particularly in the theca layer of the follicles. As previously reported, the rise in ovarian *Kiss1* mRNA at the prooestrus stage indicate a possible role for the ovarian *Kiss1* system in the regulation of ovulation [11]. This finding was also supported by differential expression of kisspeptin-IR during oestrous cycle in the Siberian hamster [12]. In the present study, post-weaning HFD exposure resulted in a marked suppression of ovarian *Kiss1* gene expression during prooestrus and oestrus at both PND 42 and PND 70, with reduced kisspeptin-IR in theca layer of antral and preovulatory follicles, suggesting that the ovarian *Kiss1* system is down-regulated under conditions of obesity and is associated with reproductive problems, such as ovulatory dysfunction. In addition, the reduction in the level of *Kiss1* expression was also observed during metoestrus at PND 70, indicating that longer HFD exposure may affect a wider range of stages. To support our hypothesisapp:addword:hypothesis, analyses in another rat model of ovulatory dysfunction induced by the administration of indomethacin exhibited a dramatic drop in ovarian *Kiss1* mRNA levels at the time of ovulation [13]. This effect was mimicked by the selective COX-2 inhibitor NS398, an inhibitor known to reduce ovulatory dynamics. Moreover, in a recent study, intraovarian administration of a kisspeptin antagonist (p234) from 22- to 50-d-old unilaterally ovariectomised rats resulted in disrupted oestrous cyclicity, and, decreased ovarian kisspeptin concentrations [30]. This study demonstrated a clear relationship between ovarian kisspeptin and successful ovulation. Although the mechanism by which ovarian kisspeptin regulate ovulatory function remains unclear, some studies have demonstrated the ability of kisspeptin to regulate MMP-2 (matrix metalloproteinase-2) and MMP-9 expression [34, 35], which are mandatory for successful ovulation, thus raising the speculation that ovarian kisspeptin may play its role in ovulation through the MMP system. In addition, our data show that the level of *GPR54* expression is not significantly changed by HFD exposure, indicating that the down-regulation of *Kiss1* mRNA and the kisspeptin protein did not affect their relative receptors.

Similar to the previous study [11], some kisspeptin/GPR54 immunostaining, not as intense as the theca cells, in the granulosa cells of antral and preovulatory follicles was detected during proestrus. Intense immunostaining was also detected in corpora lutea transformed from granulosa cells. These data raised the possibility that kisspeptin in granulosa cells may be involved in the function of the corpus luteum, which was supported by a recent study showing that the kisspeptin/GPR54 signalling system might stimulate progesterone secretion via the Erk1/2 mitogen-activated protein kinase signalling pathway in rat luteal cells [36]. However, the mechanism and physiological relevance need to be further defined. Interestingly, ovarian *Kiss1* expression may be under the positive control of gonadotropins based on the restoration by human chorionic gonadotropin [11]. Similarly, exposure of Siberian hamsters to short photoperiods induced inhibition of the HPG axis with a significant reduction in ovarian *Kiss1* expression [12]. On the other hand, local ovarian-derived kisspeptin is proposed as an ovarian factor that is involved in the modulation of gonadotropin secretion [37]. Nonetheless, sufficient experimental work will be necessary to verify such possibilities.

## Conclusions

The present study shows that exposure of female rats to a HFD had a long-term effect on reproductive function, including an earlier onset of puberty and disruption of oestrous cyclicity during both puberty and adulthood. Notably, our observations demonstrate that expression of ovarian *Kiss1* and kisspeptin was suppressed during the ovulatory transition stage under conditions of obesity, indicating that the ovarian *Kiss1* system is sensitive to obesity and may contribute to obesity-induced ovulatory dysfunction. To date, kisspeptin has been developed as a novel therapy for reproductive disorders in humans. However, clinical trials are mainly limited to studying its central stimulating effect on gonadotropin release [38, 39]. Our data call for further investigation of the potential role of the ovarian *Kiss1* system in ovulation.

## Abbreviations

AVPV: Anteroventral periventricular nucleus
ARC: Arcuate nucleus
BMP15: Bone morphogenetic protein 15
DAB: Diaminobenzidine
FSHR: Follicle stimulating hormone receptor
GDF9: Growth differentiation factor 9
GPR54: G protein-coupled receptor 54
HFD: High-fat diet
HPG: Hypothalamic-pituitary-gonadal
HPRT1: Hypoxanthine phosphoribosyltransferase 1
IR: Immunoreactivity
NCD: Normal chow diet
PND: Postnatal day
PTGS2: Prostaglandin-endoperoxide synthase 2.
